# PD-1 inhibitor treatment outcomes for cutaneous squamous cell carcinoma in patients over 85: a comparative analysis

**DOI:** 10.1093/oncolo/oyag021

**Published:** 2026-03-12

**Authors:** Ido Amir, Nofar Edri, Itamar Averbuch, Nethanel Asher, Gal Markel, Amit Ritter, Dan Yaniv, Ofir Zavdy, Gideon Bachar, Lisa Cooper, Noga Kurman, Eyal Yosefof

**Affiliations:** Department of Otorhinolaryngology and Head and Neck Surgery, Rabin Medical Center, Petah Tikva 4941492, Israel; Gray Faculty of Medicine and Health Sciences, Tel Aviv University, Tel Aviv 6997801, Israel; Department of Otorhinolaryngology and Head and Neck Surgery, Rabin Medical Center, Petah Tikva 4941492, Israel; Gray Faculty of Medicine and Health Sciences, Tel Aviv University, Tel Aviv 6997801, Israel; Gray Faculty of Medicine and Health Sciences, Tel Aviv University, Tel Aviv 6997801, Israel; Davidoff Cancer Center, Rabin Medical Center, Petah Tikva 4941492, Israel; Gray Faculty of Medicine and Health Sciences, Tel Aviv University, Tel Aviv 6997801, Israel; Davidoff Cancer Center, Rabin Medical Center, Petah Tikva 4941492, Israel; Gray Faculty of Medicine and Health Sciences, Tel Aviv University, Tel Aviv 6997801, Israel; Davidoff Cancer Center, Rabin Medical Center, Petah Tikva 4941492, Israel; Samueli Integrative Cancer Pioneering Institute, Rabin Medical Center, Petah Tikva 4941492, Israel; Department of Otorhinolaryngology and Head and Neck Surgery, Rabin Medical Center, Petah Tikva 4941492, Israel; Gray Faculty of Medicine and Health Sciences, Tel Aviv University, Tel Aviv 6997801, Israel; Department of Otorhinolaryngology and Head and Neck Surgery, Rabin Medical Center, Petah Tikva 4941492, Israel; Gray Faculty of Medicine and Health Sciences, Tel Aviv University, Tel Aviv 6997801, Israel; Department of Otorhinolaryngology and Head and Neck Surgery, Rabin Medical Center, Petah Tikva 4941492, Israel; Gray Faculty of Medicine and Health Sciences, Tel Aviv University, Tel Aviv 6997801, Israel; Department of Otorhinolaryngology and Head and Neck Surgery, Rabin Medical Center, Petah Tikva 4941492, Israel; Gray Faculty of Medicine and Health Sciences, Tel Aviv University, Tel Aviv 6997801, Israel; Gray Faculty of Medicine and Health Sciences, Tel Aviv University, Tel Aviv 6997801, Israel; Geriatric Department, Rabin Medical Center, Petah Tikva 4941492, Israel; Gray Faculty of Medicine and Health Sciences, Tel Aviv University, Tel Aviv 6997801, Israel; Davidoff Cancer Center, Rabin Medical Center, Petah Tikva 4941492, Israel; Department of Otorhinolaryngology and Head and Neck Surgery, Rabin Medical Center, Petah Tikva 4941492, Israel; Gray Faculty of Medicine and Health Sciences, Tel Aviv University, Tel Aviv 6997801, Israel; Davidoff Cancer Center, Rabin Medical Center, Petah Tikva 4941492, Israel

**Keywords:** cutaneous squamous cell carcinoma, PD-1 inhibitors, cemiplimab, elderly patients, immunotherapy outcomes

## Abstract

**Background:**

Programmed-cell death protein 1 (PD-1) inhibitors have become standard of care in the treatment of advanced or metastatic cutaneous squamous cell carcinoma (cSCC). However, insufficient data exists regarding treatment outcomes and safety among elderly patients, including patients aged >85.

**Patients and Methods:**

We retrospectively reviewed patients aged >85 (n = 52) and compared them with two control groups: 46 patients aged 71-84 and 32 patients <70, treated with cemiplimab for cSCC. Demographics, treatment characteristics, outcomes and toxicity were evaluated. Inverse probability of treatment weighting (IPTW) was applied when comparing survival outcomes.

**Results:**

Although patients >85 had worse ECOG scores and less inclined to undergo surgery, no significant differences were noted in the overall response rates (68.8% and 76.1% for <70 and 71-84, respectively vs. 73.1% for >85, *P* = .744) or disease control rates (75% and 82.6% for <70 and 71-84, respectively vs. 75% for >85, *P* = .627). After IPTW adjustment, progression-free survival (PFS) did not differ between groups (HR = 1.08, 95% CI 0.55-2.13, *P* = .82). Cancer specific survival (CSS) also did not differ between groups (HR = 1.05, 95% CI 0.22-4.87, *P* = .955). However, overall survival (OS) was shorter among patients >85 (HR = 2.64, 95% CI 1.43-4.86, *P* = .002). Toxicity profiles were similar, although toxicity-related deaths occurred more frequently in the >85 cohort.

**Conclusion:**

Cemiplimab showed comparable efficacy and tolerability across age groups, with no significant difference in PFS and CSS, but shorter OS among very elderly patients with cSCC, reflecting competing non-cancer mortality rather than reduced treatment efficacy.

Implications for Practice Cemiplimab demonstrates comparable efficacy and tolerability in patients aged >85 years with advanced or metastatic cSCC, supporting its use across age groups. The reduced overall survival in this population likely reflects competing non-cancer mortality rather than decreased treatment effectiveness. For oncology practice, these findings underscore that advanced chronological age alone should not preclude PD-1 inhibitor therapy. Incorporating comprehensive geriatric assessment, careful toxicity surveillance, and shared decision-making can optimize treatment selection and outcomes in very elderly patients, aligning immunotherapy use with individualized health status and patient-centered care principles.

## Introduction

Cutaneous squamous cell carcinoma (cSCC) is the second most common form of non-melanoma skin cancer after basal cell carcinoma, accounting for a significant portion of skin cancer-related morbidity and mortality.^1^ Owing to the fact that sun exposure is a major risk factor for cSCC, those tumors arise primarily in subsites of the head and neck region.^2^ While early-stage cSCC can often be effectively managed with surgical excision, cases of advanced or metastatic disease present a therapeutic challenge, particularly in elderly populations.^3^ The increasing incidence of cSCC in the aging population^4^ necessitates a more comprehensive understanding of both the efficacy and safety of emerging treatment modalities.

Cemiplimab, an immune checkpoint inhibitor (ICI) targeting the PD-1 pathway, has emerged as a new standard of care for patients with advanced cSCC.^5^ Clinical trials have demonstrated its efficacy in improving survival outcomes and robust response rates.^5–8^ However, limited data exists comparing the impact of age on these outcomes, especially in patients aged 85 and older, who often present with comorbidities that may affect treatment tolerance and response rates.^9^ Furthermore, there is a critical need to evaluate toxicity profiles in this age group, as it is reasonable to postulate that older and more fragile patients may experience more severe adverse effects compared to younger patients. In this context, it is worth noting that in the landmark studies conducted by Gross et al.^7^ and Midgen et al.^6^ the median age was 74 years. To date, only one randomized controlled trial assessed cemiplimab for cSCC in the adjuvant setting. In the C-POST trial,^10^ the median age was 71 years. Although no discrete >85-year subgroup was reported in the baseline data, the per-arm maximum ages (87 and 95 years) and the observed age distribution indicate that ultra-elderly patients were a small minority. Given that cSCC incidence increases sharply with age and peaks in the ninth decade and beyond,^11^ this suggests a substantial under-representation of patients >85, potentially limiting the generalizability of trial outcomes to the population most affected by the disease.

The aim of our study is to address treatment efficacy, survival outcomes and toxicity profile in the elderly patient population, with an emphasis on patients over the age of 85 years, which tends to be underrepresented in concurrent clinical trials assessing ICI therapy for cSCC.^12^

## Patients and methods

### Patients

This retrospective cohort included all cSCC patients who were treated with cemiplimab at a university-affiliated tertiary care center between 2020 and 2023. In order to maintain cohort homogeneity and ensure data completeness, patients treated with PD-1 inhibitors other than cemiplimab or patients with incomplete data were excluded.

Clinical and demographic data was collected from patients’ electronic medical records (EMR), including Eastern Cooperative Oncology Group (ECOG) performance status,^13^ primary tumor location, TNM staging based on the 8th version of the American Joint Committee of Cancer (AJCC) guidelines^14^ and prior treatment modalities.

The patients were stratified into three age groups at treatment initiation: younger than 70 years, 71 to 84 years, and 85 years or older. Comparisons were made between these groups, which allowed for adequate statistical power.

Data regarding ICI treatment was collected, including the time of treatment initiation and termination, whether ICI was used as first-line therapy, number of treatment cycles, first response to treatment, time to first response, maximal response to treatment and time to maximal response, progression on treatment, and time to progression. Response to treatment was defined as either complete response (CR), partial response (PR), stable disease (SD), or progression of disease (PD), Tumor response was assessed based on documentation in the electronic medical records (EMR), including radiology reports (one or more consecutive PET-CT scans) and the treating oncologist’s clinical assessment. Formal RECIST 1.1 criteria^15^ were not applied. Instead, responses were categorized according to the treating physician’s interpretation of radiologic findings and clinical notes, reflecting real-world practice. Objective response rate (ORR) was defined as the rate of patients with either CR or PR out of all patients in the cohort, while disease-control rate (DCR) was defined as patients with either CR, PR, or SD.

Treatment toxicity was assessed by using the grading system suggested by the Common Terminology Criteria for Adverse Events (CTCAE).^16^ Data regarding specific adverse effects was also collected and categorized into immune-related adverse events (irAE) and treatment-related adverse events (trAE). Specifically, irAEs included diarrhea, hypothyroidism, hyperthyroidism, myocarditis, nephritis, hepatitis, type 1 diabetes mellitus, adrenal insufficiency, colitis, Guillain-Barré syndrome, myasthenia gravis, and the myasthenia-myositis-myocarditis overlap syndrome. The primary outcomes of the study included overall survival (OS), progression-free survival (PFS), cancer-specific survival (CSS), and treatment efficacy metrics—maximal response, ORR, and DCR. The secondary outcome was the rate of treatment-related toxicities.

### Statistical analysis

Statistical analyses were conducted using R version 4.3.1 (R Core Team, 2023). A *P*-value threshold of 0.05 with a 95% confidence interval (CI) was used to establish statistical significance. Survival analysis was performed using the Kaplan-Meier method. Associations between categorical variables were assessed using the Fisher’s exact test. Continuous variables were compared using the Kruskal-Wallis test, as the assumption of normality was not met. The Bonferroni correction for multiple comparisons was applied as appropriate.

To adjust for baseline imbalances, inverse probability of treatment weighting (IPTW) based on propensity scores was applied. Propensity scores were estimated for age groups based on a binary distribution using logistic or gradient-boosted models (“WeighIt” package) with the following prespecified covariates: sex, ECOG group (ECOG 0, ECOG 1, ECOG ≥2), stage, nodal positivity status, and prior definitive treatment modality. Stabilized weights were used; extreme weights were examined and trimmed in sensitivity analyses. Covariate balance before and after weighting was assessed using standardized mean differences (SMDs), with SMD <0.10 considered acceptable and visualized with Love plots (“cobalt” package), included in Figure S1.

Adjusted survival curves were generated from weighted Kaplan-Meier estimators. Group comparisons underweighting were evaluated using weighted Cox proportional hazards models with robust variance estimators. Hazard ratios (HRs) and 95% CIs are reported. Proportional hazards assumptions were assessed using Schoenfeld residuals. Missing data were handled using a missing indicator approach.

## Compliance with ethical standards

### Informed consent

The study protocol received approval from the Rabin Medical Center ethics board (IRB approval number RMC-21-0515), with a waiver of informed consent.

## Results

### Patients

A total of 133 consecutive patients with cSCC treated with PD-1 inhibitors for unresectable, locally advanced, or metastatic disease in our center between 2020 and 2023 were included in the study. Of those patients, 130 (97.8%) were treated with cemiplimab, with a median follow-up duration of 11.5 months. The 3 remaining patients were treated with pembrolizumab and were excluded from the cohort. Only one patient was treated with cemiplimab with neoadjuvant intent. The most prominent subsite was the head and neck region (64.6%). The mean age at ICI treatment initiation was 79.45 years (median 80.2, IQR 18.4). The study population was stratified into three age-defined cohorts; the first group included patients <70 (n = 32), the second group included patients 71-84 (n = 46) and the third group included patients >85 (n = 52). 103 (77.44%) patients were males. The male to female ratio was not found to be different between the different age groups (corrected *P*-value = 1).

As anticipated, ECOG performance status distribution differed significantly across age groups (corrected *P*-value = .0057). Patients aged <70 and 71-84 years showed higher proportions of ECOG 0 (46.9% and 28.3%, respectively, vs. 9.6%) and ECOG 1 (37.5% and 32.6%, respectively, vs. 19.2%) compared with those aged >85 years. Conversely, a substantially higher proportion of patients aged >85 years had an ECOG performance status ≥2 (65.4%) compared with the <70 and 71-84 age groups (15.7% and 30.4%, respectively).

Tumor stage distribution was comparable across age groups, with advanced disease (stages 3 and 4) representing 43.8% of patients <70, 54.4% of patients between 71 and 84 and 51.9% of patients >85 (corrected *P*-value = 1).

Prior treatment modalities did not differ significantly between the three groups (corrected *P*-value = .211). It is, however, important to note that cemiplimab was more frequently used as the definitive treatment modality in patients aged >85, with a rate of 42.3% compared to 26.1% and 18.8% in the 71-84 and <70 groups, respectively. Correspondingly, only 36.6% of patients >85 underwent surgical treatment, with or without adjuvant therapy, versus 65.2% in the 71-84 years group and 68.8% in the <70 years group. Multimodality treatment, defined as prior surgery in combination with radiotherapy or chemoradiotherapy, was more common in the younger age groups (46.9% of patients <70 and 41.3% of patients aged 71-84). In contrast, only 21.3% of patients >85 received multimodality therapy prior to treatment with cemiplimab.

### Response to treatment

Treatment response rates did not differ between the three age groups; the overall response rate (ORR) was similar in the >85 years, 71-84 years and <70 years group, with rates of 73.1%, 76.1% and 68.8%, respectively (corrected *P*-value = 1). A similar pattern was observed in the disease control rate (DCR), with rates of 75%, 82.6%, and 75%, respectively (corrected *P*-value = 1).

The time to first treatment response (mean, in months) did not differ significantly between the groups (3.12 vs. 2.81 vs. 2.39 in the <70, 71-84 and >85 age groups, respectively, corrected *P*-value = 1). However, the time to maximal treatment response (mean, in months) was shorter in the >85 age group compared to the younger age groups, although not statistically significant (7.99 vs. 7.39 vs. 4.57 in the <70, 71-84 and >85 age groups, respectively, corrected *P*-value = .522).

Importantly, the >85 age group exhibited the lowest exposure to treatment, with a mean number of 10 cemiplimab cycles vs. 12.5 or 17 cycles in the 71-84 and <70 groups, respectively, although this difference was not shown to be statistically significant (corrected *P*-value = .132). Accordingly, treatment duration (mean, in months) was also shorter with progressing age (7 vs. 9.75 vs. 12 months in the >85, 71-84 and <70 age groups, respectively, corrected *P*-value = .704).

The complete comparison between the three age groups can be found in Table 1.

**Table 1.** Summary and comparison of demographic data and disease characteristics by age group.

	<70 (N = 32)	71-84 (N = 46)	>85 (N = 52)	*P*-value (Raw)^a^	*P*-value (Bonferroni)
**Gender**					
** Male**	27 (84.4%)	38 (82.6%)	35 (67.3%)	.103	1
** Female**	5 (15.6%)	8 (17.4%)	17 (32.7%)		
**Age at diagnosis (y)**					
** Mean (SD)**	63.06 (5.81)	75.66 (4.91)	89.59 (4.67)	<.001	<.001
** Median [IQR]**	64.6 [59.2, 67.55]	75.1 [71.825, 79.275]	89.9 [86.1, 92.875]		
**ECOG**					
** ECOG 0**	15 (46.9%)	13 (28.3%)	5 (9.6%)	<.001	.0057
** ECOG 1**	12 (37.5%)	15 (32.6%)	10 (19.2%)		
** ECOG 2**	3 (9.4%)	11 (23.9%)	17 (32.7%)		
** ECOG 3**	2 (6.3%)	3 (6.5%)	13 (25.0%)		
** ECOG 4**	0 (0%)	0 (0%)	4 (7.7%)		
** ECOG 5**	0 (0%)	0 (0%)	0 (0%)		
** Missing**	0 (0%)	4 (8.7%)	3 (5.8%)		
**Primary tumor location**					
** Face other**	6 (18.8%)	8 (17.4%)	7 (13.5%)	.188	1
** Face T zone**	2 (6.3%)	5 (10.9%)	4 (7.7%)		
** Scalp**	7 (21.9%)	13 (28.3%)	10 (19.2%)		
** Neck**	0 (0%)	1 (2.2%)	0 (0%)		
** Forearm**	0 (0%)	0 (0%)	5 (9.6%)		
** Shoulder**	2 (6.3%)	1 (2.2%)	0 (0%)		
** Hands**	0 (0%)	2 (4.3%)	1 (1.9%)		
** Trunk**	3 (9.4%)	3 (6.5%)	0 (0%)		
** Lower limb**	2 (6.3%)	0 (0%)	3 (5.8%)		
** Systemic**	0 (0%)	1 (2.2%)	0 (0%)		
** Unknown**	4 (12.5%)	8 (17.4%)	11 (21.2%)		
** Ear**	4 (12.5%)	3 (6.5%)	9 (17.3%)		
** Lips**	2 (6.3%)	1 (2.2%)	2 (3.8%)		
**T Stage**					
** Tx**	4 (12.5%)	8 (17.4%)	12 (23.1%)	.26	1
** T1**	11 (34.4%)	15 (32.6%)	8 (15.4%)		
** T2**	7 (21.9%)	11 (23.9%)	21 (40.4%)		
** T3**	8 (25.0%)	8 (17.4%)	9 (17.3%)		
** T4**	2 (6.3%)	4 (8.7%)	2 (3.8%)		
** T4a**	0 (0%)	0 (0%)	0 (0%)		
** T4b**	0 (0%)	0 (0%)	0 (0%)		
**N Stage**					
** N0**	28 (87.5%)	35 (76.1%)	34 (65.4%)	.586	1
** N1**	1 (3.1%)	2 (4.3%)	4 (7.7%)		
** N2**	0 (0%)	3 (6.5%)	4 (7.7%)		
** N2a**	0 (0%)	1 (2.2%)	2 (3.8%)		
** N2b**	1 (3.1%)	2 (4.3%)	4 (7.7%)		
** N2c**	1 (3.1%)	0 (0%)	2 (3.8%)		
** N3**	0 (0%)	3 (6.5%)	1 (1.9%)		
** N3a**	0 (0%)	0 (0%)	0 (0%)		
** N3b**	1 (3.1%)	0 (0%)	1 (1.9%)		
**M Stage**					
** M0**	30 (93.8%)	44 (95.7%)	49 (94.2%)	1	1
** M1**	2 (6.3%)	2 (4.3%)	3 (5.8%)		
**Nodal positivity status**					
** N negative disease**	28 (87.5%)	35 (76.1%)	34 (65.4%)	.0744	1
** N positive disease**	4 (12.5%)	11 (23.9%)	18 (34.6%)		
**Disease stage**					
** Stage 1**	11 (34.4%)	14 (30.4%)	8 (15.4%)	.305	1
** Stage 2**	7 (21.9%)	7 (15.2%)	17 (32.7%)		
** Stage 3**	7 (21.9%)	12 (26.1%)	13 (25.0%)		
** Stage 4**	7 (21.9%)	13 (28.3%)	14 (26.9%)		
**Prior treatment modality**					
** None**	6 (18.8%)	12 (26.1%)	22 (42.3%)	.0106	0.211
** Surgery**	7 (21.9%)	11 (23.9%)	7 (13.5%)		
** RT**	2 (6.3%)	4 (8.7%)	11 (21.2%)		
** CRT**	2 (6.3%)	0 (0%)	0 (0%)		
** Surgery + RT**	11 (34.4%)	15 (32.6%)	12 (23.1%)		
** Surgery + CRT**	4 (12.5%)	4 (8.7%)	0 (0%)		
**Number of treatment cycles (n)**					
** Mean (SD)**	16.97 (10.4)	12.52 (9.1)	10.04 (8.06)	.00695	0.132
** Median [IQR]**	16.5 [7.75, 24]	10 [5, 20]	8 [4, 13.25]		
**Treatment duration (m)**					
** Mean (SD)**	12.04 (8.96)	9.75 (7.79)	6.99 (6.16)	.037	0.704
** Median [IQR]**	10.95 [3.725, 18.35]	8.25 [3.475, 14.95]	5.6 [2.175, 8.6]		
**Time to first response (m)**					
** Mean (SD)**	3.12 (3.48)	2.81 (3.12)	2.39 (2.16)	.623	1
** Median [IQR]**	2.1 [1.175, 3.35]	2.1 [1.125, 3.175]	1.7 [1.075, 2.55]		
**Time to maximal response (m)**					
** Mean (SD)**	7.99 (6.06)	7.39 (6.73)	4.57 (3.43)	.0275	.522
** Median [IQR]**	5.55 [3.075, 13.225]	5.55 [2.325, 9.775]	3.05 [1.825, 6.65]		
**Maximal response**					
** Complete response**	13 (40.6%)	17 (37.0%)	14 (26.9%)	.504	1
** Partial response**	9 (28.1%)	18 (39.1%)	24 (46.2%)		
** Stable disease**	2 (6.3%)	3 (6.5%)	1 (1.9%)		
** Progressive disease**	8 (25.0%)	8 (17.4%)	13 (25.0%)		
**Overall response rate (ORR)**					
** SD + PD**	10 (31.3%)	11 (23.9%)	14 (26.9%)	.772	1
** CR + PR**	22 (68.8%)	35 (76.1%)	38 (73.1%)		
**Disease control rate (DCR)**					
** PD**	8 (25.0%)	8 (17.4%)	13 (25.0%)	.609	1
** CR + PR + SD**	24 (75.0%)	38 (82.6%)	39 (75.0%)		

a Association between categorical variables was assessed using the Fisher’s exact test. Association between continuous variables was assessed using the Kruskal-Wallis test.

### Survival rates

**Figure 1.**
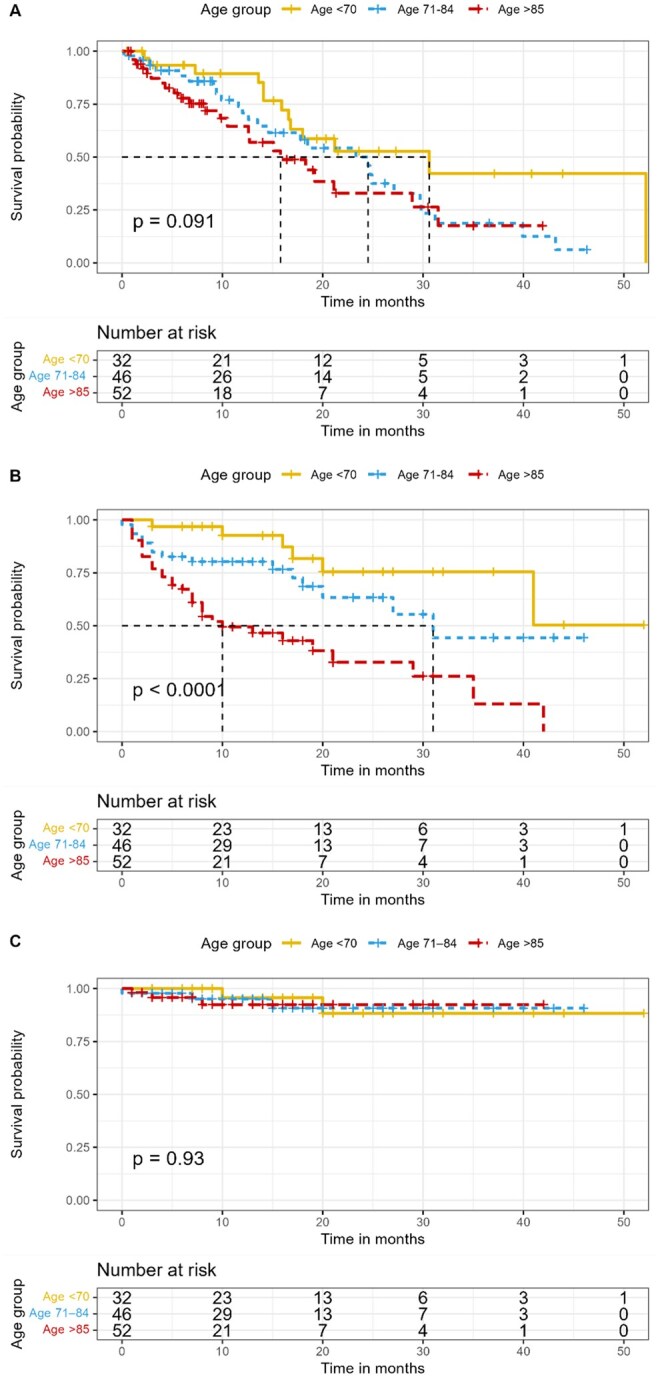
Progression-free survival (A), overall survival (B), and cancer-specific survival (C) by age-group.

Compared to younger age groups, patients older than 85 showed a trend for worse progression-free survival (Figure *1*A), with a median of 15.8 months, compared with 30.6 months for patients younger than 70 and 24.5 months for patients in the 71-84 years group. It is important to note that this difference was not found to be statistically significant (log-rank *P* = .091). No statistically significant difference (log-rank *P* = .93) was noted in cancer-specific survival (Figure 1C). However, patients older than 85 had worse overall survival (Figure 1B), with a median of 10 months, compared with 31 months in the 71-84 years group and 41 months in the younger than 70 years group (log-rank *P* < .001).

**Figure 2.**
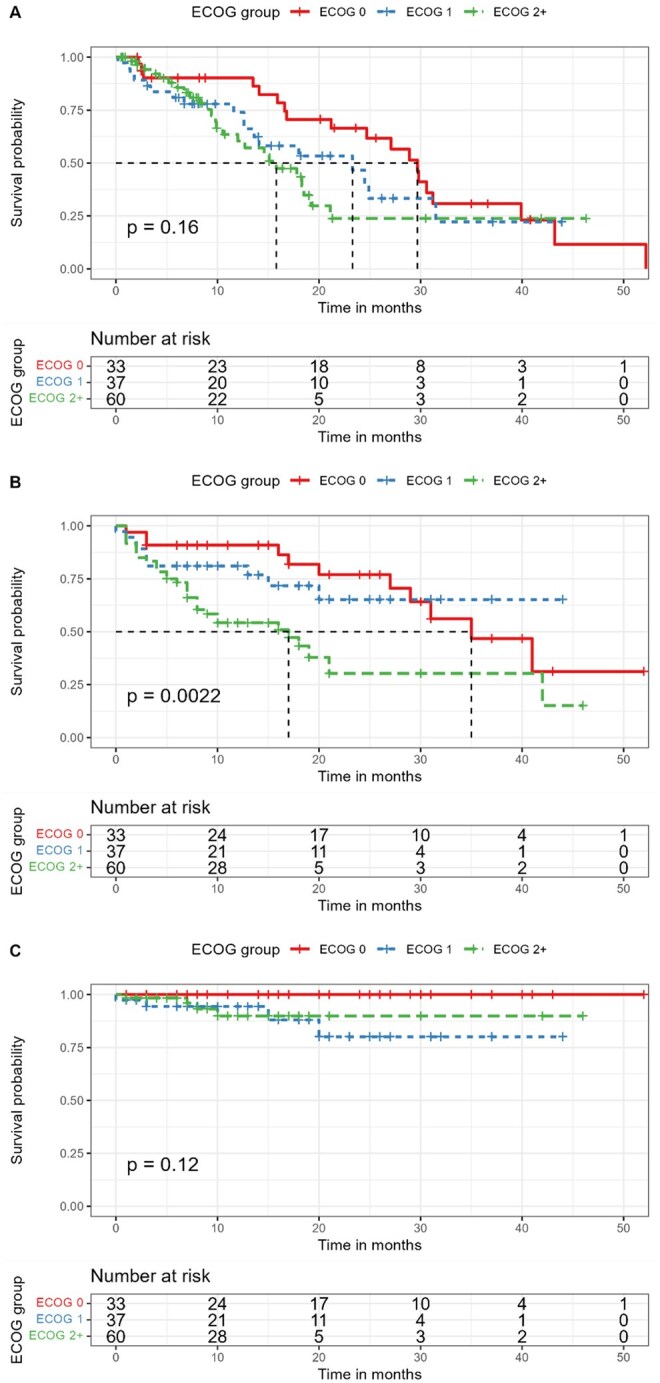
Progression-free survival (A), overall survival (B), and cancer-specific survival (C) by ECOG performance status.

A separate analysis by ECOG performance status shows a similar trend for worse progression-free survival (Figure 2A), with a median of 29.7 months for ECOG 0, 23.3 months for ECOG 1, and 15.8 months for ECOG 2 and above (log-rank *P* = .16). No statistically significant difference (log-rank *P* = .12) was noted in cancer-specific survival (Figure 2C). As expected, worse ECOG performance status corresponds to worse overall survival (Figure 2B, log-rank *P* = .0022).

### IPTW-adjusted survival outcomes

**Figure 3.**
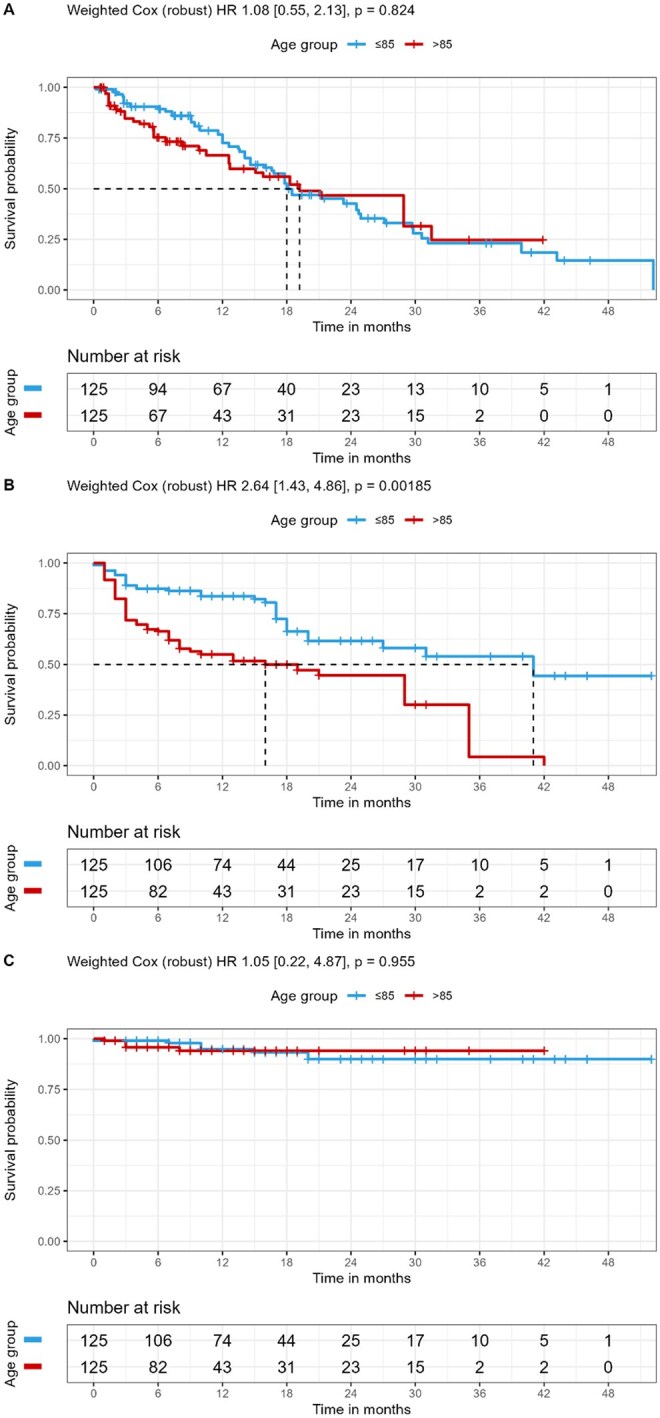
IPTW-adjusted progression-free survival (A), overall survival (B), and cancer-specific survival (C).

After weighting, the effective sample sizes were nearly identical between age cohorts (≤85: 124.6; >85: 125.2), confirming adequate covariate balance. Weighted Kaplan-Meier analyses demonstrated no significant difference in progression-free survival (weighted HR = 1.08, 95% CI 0.55-2.13, *P* = .82, Figure 3A) and cancer-specific survival (weighted HR = 1.05, 95% CI 0.22-4.87, *P* = .955, Figure 3C) between patients ≤85 and >85 years. In contrast, overall survival (OS) remained significantly shorter among patients >85 years (weighted HR = 2.64, 95% CI 1.43-4.86, *P* = .002, Figure 3B).

### Toxicity rates

The rate of trAEs in our cohort was 63.85%, and the rate of irAEs was 28.46%. There were no statistically significant differences in trAEs (65.6% vs. 63% vs. 63.5% for <70, 71-84 and >85 age groups, respectively, *P* = .881) and irAEs (31.3% vs. 26.1% vs. 28.8% for <70, 71-84 and >85 age groups, respectively, *P* = .97). No statistically significant differences were observed in toxicity rates across the different age groups (Grade 1/2, *P* = .48; Grade 3/4, *P* = .64; Grade 5, *P* = .13; Any toxicity, *P* = 1). Although the rate of grade ≥3 toxicities was highest in patients aged >85 years, the difference was not statistically significant (21.1% compared with 8.7% in the 71-84 group and 15.6% in the <70 age group, *P* = .215). While overall toxicity rates were not significantly different between age groups, patients over 85 appeared more vulnerable to severe and fatal immune-related adverse events (6 patients, 11.5%, vs. 1 patient in the 71-84 and <70 group, 2.2% and 3.1%, respectively). Specifically, 2 patients >85 died from septic shock during treatment, due to pneumonia and due to cellulitis. Neither received immunosuppressive medications. One patient succumbed to a severe systemic inflammatory response syndrome (SIRS), and another died due to suspected myocarditis. Additionally, 2 patients developed a fatal myasthenia-like syndrome; 1 of them was subsequently diagnosed with ICI-associated myasthenia gravis, myositis, and myocarditis overlap syndrome. A detailed comparison by toxicity grade can be found in Table 2. A comparison of irAE and trAE rates between the age groups, can be found in Table 3. A comprehensive list of adverse effects, along with a detailed comparison of the three age groups can be found in Table S1.

**Table 2.** Summary of toxicity grades comparison by age group.

	<70 (N = 32)	71-84 (N = 46)	>85 (N = 52)	*P*-value^a^
**Toxicity grade**				
** Grade 1/2**	16 (50.0%)	25 (54.3%)	22 (42.3%)	.48
** Grade 3/4**	4 (12.5%)	3 (6.5%)	5 (9.6%)	.64
** Grade 5**	1 (3.1%)	1 (2.2%)	6 (11.5%)	.13
**Any toxicity**	21 (65.6%)	29 (63.0%)	33 (63.5%)	1

a Association between categorical variables was assessed using the Fisher’s exact test.

**Table 3.** Rates of immune-related adverse events (irAE) and treatment-related adverse events (trAE), comparison by age group.

	<70 (N = 32)	71-84 (N = 46)	>85 (N = 52)	*P*-value^a^
**Immune-related adverse event (irAE)**				
** No**	22 (68.8%)	34 (73.9%)	37 (71.2%)	.881
** Yes**	10 (31.3%)	12 (26.1%)	15 (28.8%)	
**Treatment-related adverse event (trAE)**				
** No**	11 (34.4%)	17 (37.0%)	19 (36.5%)	.97
** Yes**	21 (65.6%)	29 (63.0%)	33 (63.5%)	

a Association between categorical variables was assessed using the Fisher’s exact test.

## Discussion

Our study, with a large cohort of patients aged 85 years or older, evaluates the treatment outcomes and adverse effects of PD-1 inhibitors in this age group, including patients with a lower performance status, and compares them with those observed in younger age groups. To the best of our knowledge, this is one of the largest comparative cohorts of patients aged 85 or more treated with PD-1 inhibitors. While the NCCN guidelines^17^ support surgery as the first treatment modality in most cases of cSCC, older or frail patients are often poor surgical candidates^18^ and may benefit from non-surgical modalities. The lower rate of surgical treatment observed among patients aged >85 years likely reflects a combination of higher nodal disease burden and poorer performance status, both of which may preclude curative-intent surgery in this population. However, importantly, even after adjustment for disease stage, nodal status, and ECOG performance status using inverse probability of treatment weighting, cancer-specific survival remained comparable across age groups, whereas overall survival remained significantly shorter in patients >85 years. These findings suggest that reduced surgical intervention in the very elderly did not translate into excess cancer-related mortality, but rather that the observed difference in overall survival is primarily driven by competing non-cancer mortality risks inherent to this age group. Taken together, these data further underscore the clinical relevance of PD-1 inhibition in the very elderly population, in whom curative-intent surgery is frequently not feasible due to frailty or comorbidity, and highlight cemiplimab as a meaningful therapeutic option capable of providing disease control without compromising cancer-specific survival.

In the context of PD-1 inhibition, aging is accompanied by immunosenescence and inflammaging—marked by reduced naïve T-cell reserves, functional exhaustion of memory T cells, and expansion of immunosuppressive cell populations, which collectively contribute to increased cancer incidence and may alter anti-tumor immunity in later life.^19^ The epidemiology of cSCC, with incidence peaking in individuals aged ≥75 years, reflects these immunologic changes, supporting the role of age-related immune dysregulation in cSCC susceptibility. Nevertheless, current clinical evidence does not indicate a consistent reduction in the efficacy of PD-1 blockade among older patients with cSCC.^20^ It is important to note that octogenarian patients were often underrepresented in the pivotal prospective studies on ICI treatment for cSCC,^5–10^ and that current knowledge on treatment effectiveness relies mostly on small retrospective studies. An observational study involving 35 patients aged 75 years or older (median 83, range 75-98) with cSCC^21^ reported a disease control rate of 91.4% and a toxicity rate of 85.7%. These findings may be attributed to favorable anti-tumoral factors, such as depleted regulatory T cell specific subpopulations within the tumor microenvironment, as evidenced in mouse melanoma models.^22^ Another small retrospective cohort by Strippoli et al.^23^ which included unselected frail patients (83.3% of the entire cohort) with cSCC (median age 81, range 36-95) found a high response rate of 76.7%, with a modest difference in ORR in favor of patients aged 81 or older (81.3% vs. 71.4% for patients <81). It is, however, difficult to deduce an unequivocal conclusion regarding treatment efficacy for our population of interest, as the authors did not disclose the exact number of patients over the age of 85. A small series^24^ of 20 cases, which specifically included patients aged 80 or older (median 86.9, range 80-103), found a response rate of 65% with a median duration of 14 months. Those findings are comparable with our data, citing a 73.1% ORR and 75% DCR. We also show a durable treatment response with a median progression-free survival of 15.8 months, despite a median OS of 10 months. The differences between OS and PFS as well as CSS could be the result of competing risks, as patients over the age of 85 are generally frailer, with lower physiological reserve, and suffer from multiple comorbidities. Thus, this may reflect higher general morbidity, rather than inferior anti-tumor activity of cemiplimab in this specific age group. As evident in our study, CSS is also generally favorable in cSCC, with rates of 93.6% at 5 and 10 years.^25^ Using an extrapolation from the United States Life Tables (2021),^26^ we can determine that the median life expectancy for the total population between the ages of 85 and 86 is 5.9 years, or 67 months. The differences between the median overall survival of our cohort of those aged >85 and the general population most probably stem from selection bias, as those patients selected for immunotherapy were generally frailer and with a more advanced disease. It should also be noted that studies in metastatic melanoma patients suggest that octogenarians achieve robust response rates with comparable tolerability to that of younger patients,^27^ consistent with our findings in elderly cSCC patients.

We also did not detect a statistically significant difference in progression-free survival according to ECOG performance status, although the median PFS was worse for ECOG 2 and above (15.8 months compared to 23.3 months and 29.7 months for ages 71-84 and <70, respectively). This is in accordance with a recent meta-analysis^28^ of 60 phase 2 and 3 randomized controlled trials showing that immunotherapy reduces the risk of death or disease progression in patients with ECOG performance status scores of 0 or 1. However, the writers also suggest that future clinical trials include more frail patients, as this population of patients was not adequately studied thus far. CSS was similar across all ECOG groups, further implying that reduced survival in ECOG ≥2 was driven primarily by competing non-cancer mortality rather than diminished response to PD-1 blockade. Expectedly, overall survival rates were shown to be significantly worse as ECOG performance status scores increased. A recent meta-analysis^29^ of real-world data on patients with non-small cell lung carcinoma (NSCLC) treated with ICI showed worse overall survival outcomes for patients with ECOG ≥2 (HR 2.72, 95% CI 2.03-3.63).

Furthermore, we demonstrated similar overall toxicity rates in the different age groups of the study. The rate of trAEs of any grade in our cohort was 63.85%, similar to the rate reported in a comprehensive meta-analysis by Wang et al.^30^ The rates of irAEs of any grade was also similar between the groups, with an overall rate of 28.46%. When looking at our data on treatment toxicity by grade, it is apparent that 78.8% of patients 85 or older experienced no adverse effects (36.5%) or grade 1 or 2 toxicities (42.3%). It is important to note that the rate of grade 3 or higher-grade toxicities was higher in the 85 or older age group (21.1% compared with 8.7% in the 71-84 group and 15.6% in the <70 age group) but was not found to be statistically significant. This is in line with data from the C-POST trial,^10^ citing a rate of 23.9% of grade ≥3 adverse events from any cause and a treatment-related rate of 9.8%. Regarding fatal toxicity (grade 5), a non-significant higher rate of fatal toxicities was demonstrated in the >85 age group. A thorough case review revealed that two of those patients suffered from myasthenia-like phenomena requiring treatment cessation and high-dose steroid treatment, with one of them subsequently diagnosed with ICI-associated myasthenia gravis, myositis and myocarditis overlap syndrome.^31^ This highlights the rationale that although ICI treatment in the octogenarian patient population is safe overall, great caution and close follow-up for any adverse reactions should be practiced. Compared with the study conducted by Denaro et al.^24^ on ultra-octogenarian patients (defined as patients aged 80 or higher), we report both a higher prevalence of treatment toxicities and a higher toxicity grade, which may be attributed to either differences in sample size or underreporting.

Our study has several limitations that should be noted. Firstly, the retrospective nature of this study may introduce selection bias, as the data might not account for all the relevant or correct variables, such as Functional Independence Measure (FIM) scores, which were not accounted for in our study due to a lack of data. Secondly, our study only included patients treated in a single tertiary center, thus limiting the number of patients available for our cohort. In addition, data on the proportion of patients requiring immunosuppressive therapy for treatment-related adverse events, as well as the underlying reasons for premature treatment discontinuation, were not available.

## Conclusion

Our study, encompassing one of the largest patient cohorts, demonstrated that treatment with cemiplimab for cSCC is both effective and well-tolerated in patients aged 85 or older, with comparable response rates to those observed in younger populations. Competing risks most likely account for differences between OS and PFS rates in patient >85. Although toxicity rates were found to be similar, lethal toxicity rates were higher in patients >85. Therefore, in patients >85, vigilant monitoring for potentially life-threatening adverse effects is warranted.

## Supplementary Material

oyag021_Supplementary_Data

## Data Availability

The data used for the preparation of this article will be shared upon reasonable request from the corresponding author.
