# Supplementary material for: PD-1 inhibitor treatment outcomes for cutaneous squamous cell carcinoma in patients over 85: a comparative analysis
Source: Oncologist. 2026 Mar 12;31(4):oyag021. doi: 10.1093/oncolo/oyag021 (PMC13006055; doi:10.1093/oncolo/oyag021)
Supplement: oyag021_Supplementary_Data [file oyag021_supplementary_data.zip › Supplementary Table S1.docx]

**Supplementary Table S1** Summary of treatment adverse effects, comparison by age group

|  | **<70 (N=32)** | **71-84 (N=46)** | **>85 (N=52)** | **P-value** |
| --- | --- | --- | --- | --- |
| **Any toxicity** |  |  |  |  |
| No | 11 (34.4%) | 17 (37.0%) | 19 (36.5%) | 1 |
| Yes | 21 (65.6%) | 29 (63.0%) | 33 (63.5%) |  |
| **Pruritus** |  |  |  |  |
| No | 26 (81.3%) | 35 (76.1%) | 42 (80.8%) | 0.855 |
| Yes | 6 (18.8%) | 11 (23.9%) | 10 (19.2%) |  |
| **Fatigue** |  |  |  |  |
| No | 27 (84.4%) | 33 (71.7%) | 35 (67.3%) | 0.219 |
| Yes | 5 (15.6%) | 13 (28.3%) | 17 (32.7%) |  |
| **Rash** |  |  |  |  |
| No | 29 (90.6%) | 40 (87.0%) | 47 (90.4%) | 0.878 |
| Yes | 3 (9.4%) | 6 (13.0%) | 5 (9.6%) |  |
| **Diarrhea** |  |  |  |  |
| No | 30 (93.8%) | 43 (93.5%) | 47 (90.4%) | 0.841 |
| Yes | 2 (6.3%) | 3 (6.5%) | 5 (9.6%) |  |
| **Hyperthyroidism** |  |  |  |  |
| No | 31 (96.9%) | 46 (100%) | 52 (100%) | 0.246 |
| Yes | 1 (3.1%) | 0 (0%) | 0 (0%) |  |
| **Arthralgia** |  |  |  |  |
| No | 32 (100%) | 45 (97.8%) | 48 (92.3%) | 0.296 |
| Yes | 0 (0%) | 1 (2.2%) | 4 (7.7%) |  |
| **Anemia** |  |  |  |  |
| No | 32 (100%) | 46 (100%) | 51 (98.1%) | 1 |
| Yes | 0 (0%) | 0 (0%) | 1 (1.9%) |  |
| **Nausea** |  |  |  |  |
| No | 32 (100%) | 44 (95.7%) | 50 (96.2%) | 0.677 |
| Yes | 0 (0%) | 2 (4.3%) | 2 (3.8%) |  |
| **Hypothyroidism** |  |  |  |  |
| No | 31 (96.9%) | 41 (89.1%) | 49 (94.2%) | 0.457 |
| Yes | 1 (3.1%) | 5 (10.9%) | 3 (5.8%) |  |
| **Pneumonitis** |  |  |  |  |
| No | 30 (93.8%) | 43 (93.5%) | 50 (96.2%) | 0.791 |
| Yes | 2 (6.3%) | 3 (6.5%) | 2 (3.8%) |  |
| **Dermatitis** |  |  |  |  |
| No | 30 (93.8%) | 44 (95.7%) | 48 (92.3%) | 0.897 |
| Yes | 2 (6.3%) | 2 (4.3%) | 4 (7.7%) |  |
| **Arthritis** |  |  |  |  |
| No | 31 (96.9%) | 45 (97.8%) | 52 (100%) | 0.516 |
| Yes | 1 (3.1%) | 1 (2.2%) | 0 (0%) |  |
| **Adrenal insufficiency** |  |  |  |  |
| No | 32 (100%) | 45 (97.8%) | 51 (98.1%) | 1 |
| Yes | 0 (0%) | 1 (2.2%) | 1 (1.9%) |  |
| **Collitis** |  |  |  |  |
| No | 31 (96.9%) | 46 (100%) | 51 (98.1%) | 0.714 |
| Yes | 1 (3.1%) | 0 (0%) | 1 (1.9%) |  |
| **Hepatitis** |  |  |  |  |
| No | 31 (96.9%) | 45 (97.8%) | 51 (98.1%) | 1 |
| Yes | 1 (3.1%) | 1 (2.2%) | 1 (1.9%) |  |
| **Myocarditis** |  |  |  |  |
| No | 32 (100%) | 45 (97.8%) | 51 (98.1%) | 1 |
| Yes | 0 (0%) | 1 (2.2%) | 1 (1.9%) |  |
| **Nephritis** |  |  |  |  |
| No | 32 (100%) | 46 (100%) | 51 (98.1%) | 1 |
| Yes | 0 (0%) | 0 (0%) | 1 (1.9%) |  |
| **Myasthenia gravis** |  |  |  |  |
| No | 32 (100%) | 46 (100%) | 51 (98.1%) | 1 |
| Yes | 0 (0%) | 0 (0%) | 1 (1.9%) |  |
| **Gullian-barre syndrome** |  |  |  |  |
| No | 32 (100%) | 45 (97.8%) | 52 (100%) | 0.6 |
| Yes | 0 (0%) | 1 (2.2%) | 0 (0%) |  |
| **Weight loss** |  |  |  |  |
| No | 30 (93.8%) | 46 (100%) | 50 (96.2%) | 0.23 |
| Yes | 2 (6.3%) | 0 (0%) | 2 (3.8%) |  |
| **Ageusia** |  |  |  |  |
| No | 32 (100%) | 45 (97.8%) | 51 (98.1%) | 1 |
| Yes | 0 (0%) | 1 (2.2%) | 1 (1.9%) |  |
| **Type 1 diabetes mellitus** |  |  |  |  |
| No | 32 (100%) | 46 (100%) | 51 (98.1%) | 1 |
| Yes | 0 (0%) | 0 (0%) | 1 (1.9%) |  |
| **Cytopenia** |  |  |  |  |
| No | 31 (96.9%) | 44 (95.7%) | 50 (96.2%) | 0.465 |
| Yes | 1 (3.1%) | 2 (4.3%) | 2 (3.8%) |  |
| **Fever** |  |  |  |  |
| No | 31 (96.9%) | 46 (100%) | 52 (100%) | 0.246 |
| Yes | 1 (3.1%) | 0 (0%) | 0 (0%) |  |
| **Psoriasis** |  |  |  |  |
| No | 30 (93.8%) | 46 (100%) | 51 (98.1%) | 0.253 |
| Yes | 2 (6.3%) | 0 (0%) | 1 (1.9%) |  |
| **Raynaud’s phenomenon** |  |  |  |  |
| No | 31 (96.9%) | 46 (100%) | 52 (100%) | 0.246 |
| Yes | 1 (3.1%) | 0 (0%) | 0 (0%) |  |
| **Weakness** |  |  |  |  |
| No | 30 (93.8%) | 45 (97.8%) | 49 (94.2%) | 0.655 |
| Yes | 2 (6.3%) | 1 (2.2%) | 3 (5.8%) |  |
| **Other** |  |  |  |  |
| No | 30 (93.8%) | 42 (91.3%) | 45 (86.5%) | 0.57 |
| Yes | 2 (6.3%) | 4 (8.7%) | 7 (13.5%) |  |
| **Neuropathy** |  |  |  |  |
| No | 30 (93.8%) | 46 (100%) | 52 (100%) | 0.059 |
| Yes | 2 (6.3%) | 0 (0%) | 0 (0%) |  |
| **Myasthenia-myocarditis-myositis overlap syndrome** |  |  |  |  |
| No | 30 (93.8%) | 46 (100%) | 51 (98.1%) | 0.254 |
| Yes | 2 (6.3%) | 0 (0%) | 1 (1.9%) |  |

* Association between categorical variables was assessed using the Fisher’s exact test.
