# Supplementary figures and images for: PD-1 inhibitor treatment outcomes for cutaneous squamous cell carcinoma in patients over 85: a comparative analysis
Source: Oncologist. 2026 Mar 12;31(4):oyag021. doi: 10.1093/oncolo/oyag021 (PMC13006055; doi:10.1093/oncolo/oyag021)

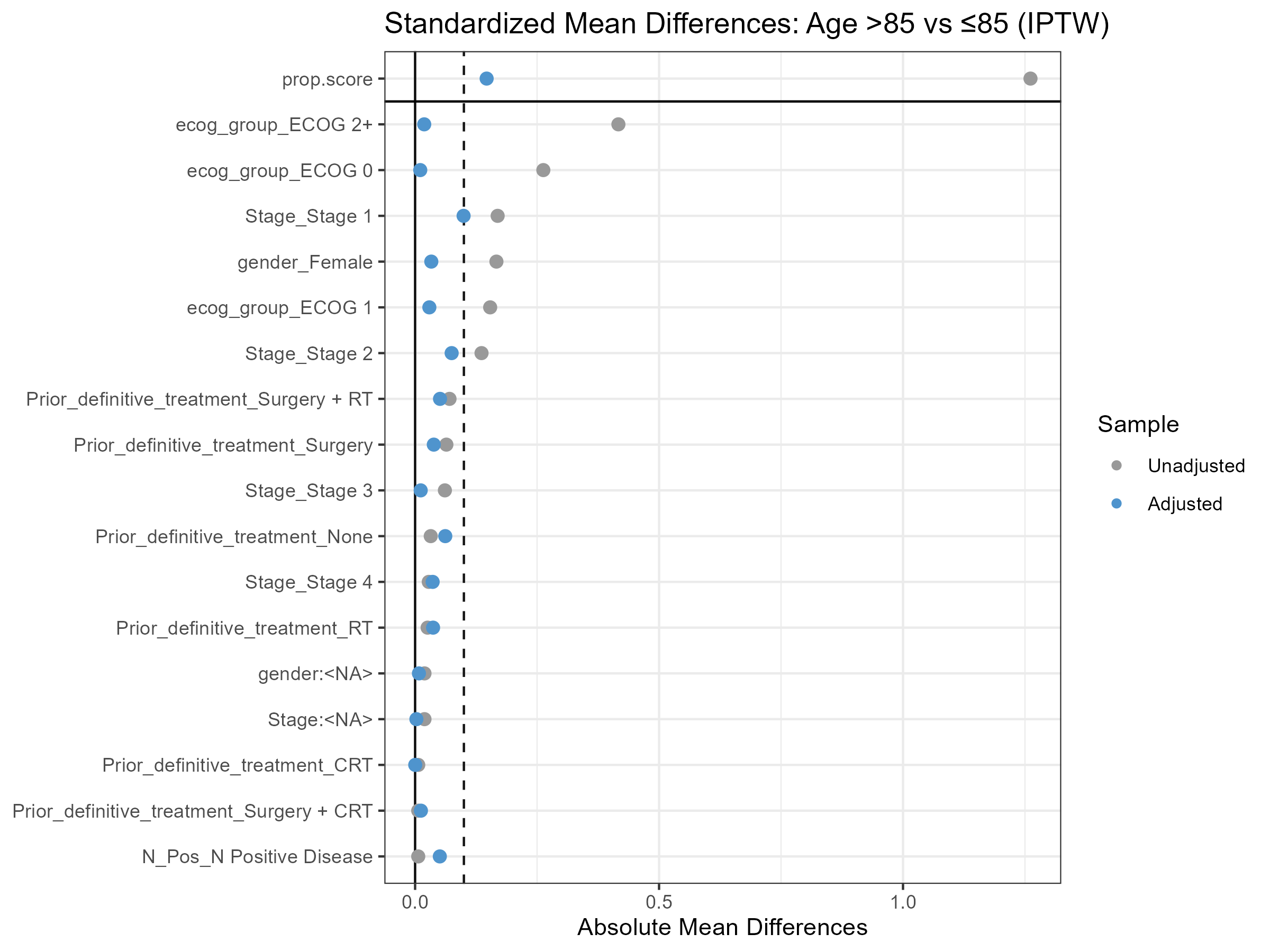

Supplement: oyag021_Supplementary_Data [file oyag021_supplementary_data.zip › Supplementary Figure S1.png]
